# Case of Diffused Aneurism

**Published:** 1831-04-01

**Authors:** 


### GRAMPUS HOSPITAL SHIP.

#### DIFFUSED ANEURISM.

MR. BENNETT, assistant surgeon of the Grampus, has succinctly and clearly reported a case of the above disease, in No. 374, of the Lancet. With the omission of a few unimportant passages, we give the principal part of the case in the words of the reporter.

*Case.* "John Morgan, ætat. 32, seaman, was admitted on board this hospital on the 28th Sept. 1830. His appearance emaciated and exsanguine; his countenance sallow and anxious. He complained of pain in the situation of a tumour, about fourteen inches in circumference, occupying the lower third and inner side of the *right* thigh, presenting its greatest bulk in that situation, and gradually decreasing forwards to the ham and outer side of the thigh. The circumference of the tumour had a defined, hardened, margin; the summit was tense, elastic, and gave to the touch an evident sense of fluctuation; the integuments retained their natural colour; the leg was somewhat œdematous, and remained flexed, without the power of extension. The patient denied that any morbid appearance, or uneasiness, had existed in the part previous to seven weeks since, when, without assignable cause, the whole leg and foot became swelled and tense, and ultimately 'settled' into the present tumour. Upon the most careful examination no pulsation could be detected in the enlarged surface, except to a slight degree in that position which lay immediately over the seat of the popliteal artery. Pressure obstructing the passage of the blood through the femoral artery produced no diminution in the bulk of the tumour, nor could any sound be detected upon application of the stethoscope. The patient had no recollection of any pulsating tumour having ever appeared in the ham. He had had some severe rigors, and evidently suffered much constitutionally. Under these doubtful circumstances the limb was placed on a pillow, resting on its outer side, and the evaporating lotion applied. On the 29th, the tumour being in no way diminished, but rather more tense, the necessity became apparent of settling the question whether the tumour was occasioned by a collection of matter, or by disease of a more serious character. Preparations were, therefore, made to secure the femoral artery, should the tumour prove aneurismal upon introducing a lancet into its substance. This was done to the depth of an inch in a valvular direction, but with no other result than the escape of a few drops of dark blood. This attempt to ascertain the true nature of the disease having failed, adhesive plaster was placed over the puncture, and the limb restored to a state of rest, without any appearance of a disposition to hæmorrhage.

On the 30th, at noon, the patient having moved the limb roughly, and placed it over the side of his bed, about an ounce of fluid blood, unattended by arterial jet, passed from the aperture made in the tumour the day previous, and was easily checked by placing the limb in a quiet position, and by slight pressure with lint. The two following days were passed without any change in the appearance of the tumour, with the exception of a slight apparent extension towards the upper part of the thigh. A probe introduced into the lancet-orifice in the tumour, passed its entire length in every direction without resistance and without hæmorrhage.

On the 3d Oct. the swelling had extended considerably up the thigh, occupying its lower half, was much more tense at its original seat, and gave a more evident sense of fluctuation immediately above the patella. Under these circumstances, Dr. Dobson, principal surgeon to Greenwich Hospital, who attended in consultation on the case, declared his opinion, that, taking into consideration the enfeebled powers of the patient, and the mass of disease in which the parts were evidently involved, the removal of the limb gave the only chance of recovery to the patient; and he having given his consent, amputation was performed on the same day by Mr. Bennett, assistant-surgeon to the hospital, in the presence of Dr. Dobson and Mr. Gilchrist. The limb was removed at the upper third of the thigh by the circular incision; about eight or ten ounces of blood were lost during the operation; ligatures were placed on the femoral, profunda, and three minor arteries."

After some vicissitudes of rallying and exhaustion, he sunk the next day (4th) at eleven o'clock at night.

*Dissection.* "Upon examination of the removed limb, between two and three pints of dark blood, partly coagulated and partly fluid, were found occupying the lower half of the thigh, nearly insulating the lower third of the shaft of the femur, which to the extent of four inches was denuded of periosteum, and presented a honeycomb appearance. The chief volume of effused blood occupied the situation of the muscles (which were nearly absorbed), and in many parts was in contact with the integuments, chiefly so immediately above the patella. Upon pursuing the examination, a fine aneurismal sac, about the size of a pullet's egg, evidently formed by a dilatation of the three structures of the artery, was found on the anterior surface of the popliteal artery; the sac, at its upper third and anterior surface, was rent to the extent of two inches in a transverse direction. Immediately above the torn sac, and externally, appearing to form a portion of it, was a second dilatation of the artery forming a sac, the size of a small walnut, lined with a thick layer of coagulum, and communicating with the larger and torn sac by an opening in size not exceeding a third of the natural calibre of the femoral artery. The cellular tissue of the leg and foot was loaded with serum."

The heart appears to have been in a state of passive dilatation; and the "*right* kidney was placed immediately over the common iliac vessels of the same side."

